# Analysis of laser radiation using the Nonlinear Fourier transform

**DOI:** 10.1038/s41467-019-13265-4

**Published:** 2019-12-11

**Authors:** Srikanth Sugavanam, Morteza Kamalian Kopae, Junsong Peng, Jaroslaw E. Prilepsky, Sergei K. Turitsyn

**Affiliations:** 1 Aston Institute of Photonic Technologies, Aston University, Aston Triangle, Birmingham, B4 7ET UK; 2 State Key Laboratory of Precision Spectroscopy, East China Normal University, Shanghai, 200062 China; 3 Aston-Novosibirsk International Centre for Photonics, Novosibirsk State University, Novosibirsk, 630090 Russia

**Keywords:** Fibre optics and optical communications, Fibre lasers, Mode-locked lasers, Nonlinear optics

## Abstract

Modern high-power lasers exhibit a rich diversity of nonlinear dynamics, often featuring nontrivial co-existence of linear dispersive waves and coherent structures. While the classical Fourier method adequately describes extended dispersive waves, the analysis of time-localised and/or non-stationary signals call for more nuanced approaches. Yet, mathematical methods that can be used for simultaneous characterisation of localized and extended fields are not yet well developed. Here, we demonstrate how the Nonlinear Fourier transform (NFT) based on the Zakharov-Shabat spectral problem can be applied as a signal processing tool for representation and analysis of coherent structures embedded into dispersive radiation. We use full-field, real-time experimental measurements of mode-locked pulses to compute the nonlinear pulse spectra. For the classification of lasing regimes, we present the concept of eigenvalue probability distributions. We present two field normalisation approaches, and show the NFT can yield an effective model of the laser radiation under appropriate signal normalisation conditions.

## Abstract (web summary)

Mathematical approaches for simultaneous characterisation of localized and extended fields in optical signals are not well developed. Here, the authors demonstrate the application of the Nonlinear Fourier transform approach based on the Zakharov-Shabat spectral problem for the analysis of fibre laser radiation.

## Introduction

Characterisation and processing of complex temporal signals are critically important for a wide range of applications, from optical communications to medicine. It is well known that classical signal processing methods, such as the Fourier transform (FT) are not always well suited for analysis of dynamically changing, non-stationary signals. This is because these methods implicitly assume stationarity of the considered fields. For instance, the traditional FT synthesizes signal through a linear combination of time-invariant (stationary), lasting-to-infinity sinusoids. In general, a non-stationary signal cannot be efficiently represented using a linear combination of stationary signals. For analysis of such signals of aperiodic nature (for instance pulses limited both in time and frequency) different mathematical tools like windowed FT, nonlinear time series analysis, etc. have been developed and successfully used^1–4^. However, methods for systematic analysis of signals comprising both dispersive waves and structures localised in time are less developed. Here, we introduce technique based on the so-called nonlinear Fourier transform (NFT) for characterisation of fibre laser radiation that presents a mixture of dispersive waves and optical pulses.

Fibre laser technology is an active field of research and development, and finds a broad range of applications, see for e.g.^5–8^ and references therein. Beyond their very evident practical importance, fibre lasers are also interesting from the standpoint of physics as test-beds for nonlinear wave dynamics^9–18^. Obtaining a deeper insight into the nonlinear dynamics and instabilities in fibre lasers is a promising opportunity for unlocking new lasing regimes with unusual and potentially superior characteristics^19–21^.

The challenge lies in the experimental study and classification of such nonlinear operational regimes. Conventional techniques and apparatuses like radio-frequency and optical spectrum analysers, and others are not suited for capturing rapid non-repetitive laser output field or even intensity variations. To tackle this technological hurdle, several advanced characterisation methods have been proposed and demonstrated (see, e.g. refs. ^22–25^), which have opened a new frontier in ultra-fast measurement technology for unveiling transient phenomena in nonlinear laser dynamics. For instance, the dispersive Fourier transform (DFT) method for real-time spectral measurement^22^ has been successfully used in fibre lasers for studying soliton explosions^26^, bound solitons^24,27^, and the complexities of mode-locking onset behaviour^28^. Further, measurements of spatio-temporal dynamics of intensity in fibre lasers^29,30^ have revealed the existence of distinct spatio-temporal regimes of operation in ultralong fibre lasers^29,31^, proliferation of dark solitons in stretched pulse fibre lasers^29^, generation of spatio-temporal extreme events^32^ and also interaction and collisions of solitons in multisoliton regimes^33^. The methodology of space-time duality^34^ has been successfully exploited to realise time lenses for the study of integrable turbulence^35^, the direct observation of rogue waves^36^ and in uncovering hidden universalities in soliton dynamics^37^.

Several of the operational regimes highlighted above possess a common characteristic, wherein localised (in time) structures interact with background dispersive waves. Currently, the description of such operational regimes using a single, self-consistent formalism, which is clearly able to discriminate between these two radiation components and unravel their interaction dynamics is lacking.

Here we examine application of the NFT approach based on the Zakharov-Shabat spectral problem for description of laser radiation with a mixture of pulses and dispersive waves. Also known as the inverse scattering transform, the NFT has been extensively used for description of solutions of the integrable nonlinear systems, including analysis of nonlinear propagation of light in optical fibres^38–42^, and also in other disciplines where integrable models occur, for instance, for description of the oceanic waves^43^. More recently, the use of the NFT has also been applied for analysis of laser radiation^44^. However, key questions about the conceptual underpinnings of the applicability of the NFT for analysis of dissipative, non-integrable systems still remain.

This work contributes new knowledge to this emerging field. We explicate how the NFT can be used for analysis of laser radiation that contains pulses embedded in dispersive waves. We proffer that much like the conventional FT which decomposes analysed radiation into an orthogonal basis of Fourier eigenmodes, the NFT based on the Zakharov-Shabat spectral problem can be employed as a signal processing tool that decomposes signals into a basis made up by the localised (solitary) eigenmodes and nonlinear dispersive radiation components.

We would like to stress that the NFT is not used here for solving any underlying equations, as in the theory of the integrable systems where it was originally developed, but rather exploited as a signal processing tool. To better understand this, our approach can be compared to the use of the classical linear FT in nonlinear systems for obtaining spectra of the evolving field. Evolution of spectral FT components in this case is not trivial as the spectral modes interact with each other. Yet, this linear decomposition still provides useful information for scientists and engineers, e.g. via the identification of harmonics. In the case of using the NFT based on the Zakharov-Shabat spectral problem for signal processing of localised waveforms one can expect pulses to be approximated by a limited number of discrete eigenvalues, simplifying description compared to use of a large number of linear spectral harmonics. NFT components can be computed with round-trip time resolution, revealing the characteristic distribution of embedded localised modes and respective real-time pulse evolution features.

In this regard, we address the important question of normalisation. When calculating the NFT, different choices of normalisation parameters will lead to different distributions of energy between nonlinear spectral components. The purpose of choosing the right normalisation convention is to arrive at the minimal possible number of the degrees of freedom required to adequately describe the laser radiation. To this end, we present here two normalisation strategies. The first entails normalising the system using the averaged fibre parameters, similar to ref. ^44^. The second approach reduces the initial system to effective evolution of a single localised nonlinear mode. This then is used to demonstrate how the NFT can be effectively used for the derivation of approximate models for describing pulse evolution in lasers, an inherently non-integrable system. This approach can be considered as a version of the Galerkin approximation method that allows under certain conditions the reduction of a continuous problem with infinite degrees of freedom to a discrete problem with finite number of parameters.

## Results

### Basics of NFT decomposition

The nonlinear Schrödinger equation (NLSE), including its modifications, is the classical model that is applied to the description of light propagation in optical fibres. In 1972 Zakharov and Shabat demonstrated that the initial-value problem for the NLSE, governing the propagation along the $z$-coordinate of a complex envelope function of time, $q(t,z)$,

1 $$i\frac{\partial q(t,z)}{\partial z}+\frac{{\partial }^{2}q(t,z)}{\partial {t}^{2}}+2| q(t,z){| }^{2}q(t,z)=0,$$

can be solved in a manner very similar to the solution of linear evolutionary equations by using the inverse scattering transform technique^38^ (often referred nowadays to as the NFT in the optical signal processing literature^45^). The NFT operations transform the localised time-domain pulse onto some special basis, the nonlinear Fourier (NF) spectrum. In this form, description of the field evolution in Eq. (1) becomes trivial and similar to linear propagation. Here we use term pure NLSE for Eq. (1) to distinguish it from its perturbed variant to be discussed later.

The NFT decomposition of a given pulse $q(t)$ is defined in terms of solutions of the so-called Zakharov-Shabat spectral problem, written for two auxiliary functions ${v}_{1,2}$^38^

2 $$\frac{\mathrm{d}}{{\mathrm{d}}t}\left(\begin{array}{c}{v}_{1}(t,\zeta )\\ {v}_{2}(t,\zeta )\end{array}\right)=\left(\begin{array}{ll}-i\zeta &q(t)\\ -{q}^{* }(t)&i\zeta \end{array}\right)\left(\begin{array}{l}{v}_{1}(t,\zeta )\\ {v}_{2}(t,\zeta )\end{array}\right),$$

where $q(t)$ is the signal to process, decaying at the asymptotic limits $t\to \mp \infty$ (we drop its dependence on $z$), and $\zeta$ is the spectral parameter which can be understood as a nonlinear analogue of conventional Fourier frequency. The asterisk in Eq. (2) and below denotes complex conjugates of corresponding quantities. To retrieve the spectral data associated with the given profile $q(t)$, we fix the so-called Jost solution $\Phi (t,\zeta )$ of Eq. (2) imposing the asymptotic condition:

3 $$\Phi (t,\zeta )\equiv \left(\begin{array}{l}{\phi }_{1}\\ {\phi }_{2}\end{array}\right) \mathop{{----\to}}\limits_{t\to -\infty }\left(\begin{array}{l}{e}^{-i\zeta t}\\ 0\end{array}\right).$$

The goal of the NFT pulse decomposition is to find the continuous and discrete spectral quantities associated with $q(t)$. The spectral quantities $a(\zeta )$ and $b(\zeta )$, defined through the special solution $\Phi (t,\zeta )$ as

4 $$a(\zeta )=\mathop{\mathrm{lim}}\limits_{t\to +\infty }{\phi }_{1}(t,\zeta ){e}^{i\zeta t},\qquad b(\zeta )=\mathop{\mathrm{lim}}\limits_{t\to +\infty }{\phi }_{2}(t,\zeta ){e}^{-i\zeta t},$$

play the central role in the transform. The continuous part of nonlinear Fourier spectrum (the incoherent waves) is given by the ratio $r(\zeta )=b(\zeta )/a(\zeta )$ for real ${\zeta }$, where $r(\zeta )$—the so-called reflection coefficient—plays the role of the ordinary Fourier spectrum and converges to it in the low-power limit. The solitonic degrees of freedom (the coherent part of nonlinear Fourier spectrum) are associated with the discrete spectral data consisting of the set of complex-valued eigenvalues $\{{\zeta }_{j}\}$ of Eq. (2) having a positive imaginary part. The solution and analysis of those ${\zeta }_{i}$ is done in our current work. The second part of discrete NFT spectrum consists of complex-valued norming constants $\{{c}_{j}\}$, associated with each eigenvalue; however these quantities are not used in our current study. For more mathematical details see^38,45^ and Supplementary Note 2.

For the pure NLSE solutions decaying to zero at $t\to \pm\! \infty$, i.e. for temporally-localised optical waveforms, the nonlinear spectrum consists of two parts as mentioned above—the discrete eigenvalues and the continuous spectrum. When considering the pure NLSE (1), each component of that pair evolves independently of other modes without nonlinear cross-talk, i.e. stays orthogonal to the rest of the pulse content, again, similarly to the behaviour of orthogonal Fourier harmonics in linear systems.

Complex eigenvalues—the complex-valued nonlinear spectral parameters from Eq. (2) describing the embedded solitary modes^45^—can be understood as the complex frequencies corresponding to each individual solitary NF mode. Notably, in the pure NLSE (1) the complex eigenvalues stay invariant during the pulse evolution. Thus, by analysing the NF spectrum of the evolving pulse we can readily segregate the continuous spectrum that represents the dispersive component of the pulse from the complex eigenvalue spectrum that refers to its invariant or coherent, solitary components^46^. Indeed, the NFT was applied recently in the so-called integrable turbulence to monitor the appearance of coherent structures, such as breathers, solitons and rogue waves^47,48^.

The exact orthogonality of nonlinear eigenmodes and the invariance of complex eigenvalues are broken when the true dynamical system differs from the pure NLSE (1). Such a scenario can be envisioned when Eq. (1) incorporates a term to account for intrinsic fibre material losses, or equivalently, gain as in the case of lasers. This transforms the equation from an integrable to a non-integrable one. However even in this case, one can formally calculate nonlinear spectrum using the Zakharov-Shabat spectral problem at each point in $z$ and trace the evolution of discrete eigenvalue parameters and the continuum spectrum with distance. In fact, the robustness of the NFT signal components due to the strong influence of the NLSE core is the momentous feature utilised in the recently emerged NFT-based communication systems^45^, where the underlying evolution can also differ significantly from that governed by the unperturbed NLSE.

In other words, even though the NFT based on the Zakharov-Shabat spectral problem was initially developed as a method for the solution of nonlinear integrable equations, it can still be used as a signal processing method to represent the complex field in a convenient basis. We would like to reiterate that conceptually our approach is similar to the one utilising linear FT to analyse inherently nonlinear signals, representing them in an orthogonal sinusoidal basis. While the latter allows the signal to be represented as a sum of sinusoids, i.e. the linear Fourier eigenmodes, this is just a signal decomposition, and more complex alternative techniques like mutual information analysis^49^ are required to reveal the underlying nonlinear correlations between the different spectral components. In contrast, the robustness of the NLSE core equips us with the additional framework to monitor the pulse evolution, being specifically convenient in the scrutiny of coherent part of the signal due to the inherent capability of NFT to discern the localised and dispersive constituents^46^. Indeed, the NFT approach was applied with this notion very recently in analysing single-shot mode-locked pulses in fibre lasers^44^, and for a specific laser system modelled using the NLSE with saturated gain, dissipative nonlinear and dispersive terms^50^.

In the following, we show in more detail how the NFT can be used for the real-time monitoring of signal in the inherently dissipative and non-integrable system such as the mode-locked laser. Specifically, we show that the coherent features revealed by the NFT map one-to-one to localised structures observed in the laser intensity spatio-temporal dynamics, how this mapping can be used to track the appearance of localised structures in real-time, use it for representation and classification of lasing regimes as eigenvalue distribution functions, and under certain conditions how the NFT can be used to derive an approximate model for the laser.

### Real-time NFT discrete eigenvalue spectrum

NFT-based signal processing requires full-field information, i.e., the knowledge of both the power (or intensity) and the phase. Note that while methods like spatio-temporal dynamics^29,30^ and DFT^22^ are often used for studying the dynamic behaviour of laser field, they do not capture phase information. The bottleneck lies in the intensity-based response of the square law detectors which average out all fast phase variation. Several methods for optical phase recovery have been demonstrated to fill this deficiency, for instance by use of iterative phase-error minimisation algorithms^44,51^, specially designed gratings^52^, spectral interferometry^53^, or even a combination of the aforementioned techniques as demonstrated recently^54^.

**Fig. 1:**
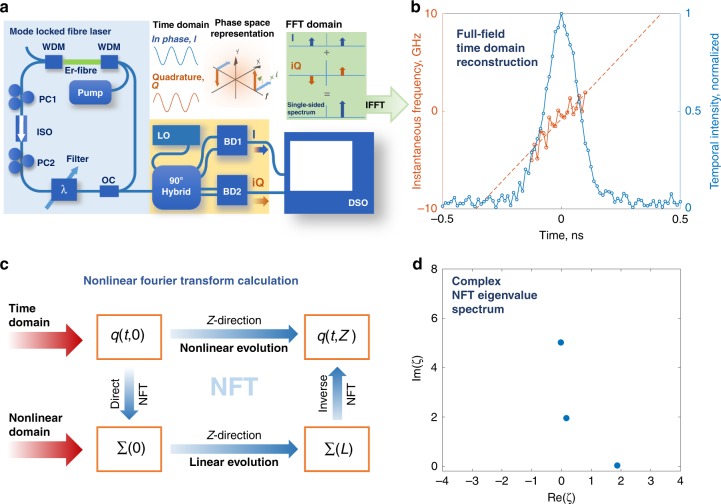
Calculating the NFT spectrum of a mode-locked pulse. **a** Experimental configuration. The blue shaded box shows the mode-locked fibre laser used in the experiment. Er—Erbium; WDM—wavelength division multiplexer; PC1, PC2—Polarization controllers; ISO—Isolator; OC—output coupler; LO—Local oscillator; BD1, BD2—Balanced detectors, DSO—Digital storage oscilloscope. The yellow shaded box shows the real-time coherent homodyne detection configuration; the $9{0}^{0}$ hybrid generates in-phase and quadrature copies of the laser signal which aids in reconstruction of the single-sided complex-valued spectrum, as shown using the phase space and FFT domain representation. **b** Full field reconstruction of the mode-locked pulse in the time domain obtained from the inverse FFT of the single-sided spectrum from (**a**). **c** Flow diagram showing the relation between the physical and NFT domains. The complex time-domain signal is fed into the NFT signal processing algorithm to yield its nonlinear decomposition. **d** The discrete NF spectrum of the pulse in (**a**).

Here we demonstrate the NFT signal processing methodology using experimentally measured real-time full-field dynamics of mode-locked laser pulses obtained using coherent homodyne detection^55–58^. Figure 1a shows the unidirectional erbium gain-based ring-fibre laser used for generation of the mode-locked pulses. The length of the laser cavity is 13 m. The lasing threshold is observed at a current value of 600 mA. The laser is mode-locked using the mechanism of nonlinear polarization evolution. A Gaussian intra-cavity filter with adjustable spectral location and bandwidth is used to control the spectral width of generation. The laser gives rise to stretched Gaussian pulses of width $\sim$200 ps, centred about 1555 nm, with spectral bandwidth of 0.11 nm. While the fibre is all anomalous, the slightly normal Er-fibre and the use of bulk optics in the adjustable filter results in the fibre laser giving rise to stretched pulses. It operates in the fundamental mode-locked regime for the whole range of measurements presented in this paper, giving rise to chirped pulses that can be approximated by the Gaussian or sech-type shape.

To implement homodyne detection, the laser output is mixed with a narrow-linewidth laser and fed to a passive optical network called the $9{0}^{0}$ hybrid, which gives rise to an in-phase (I) and the quadrature (Q) component. These outputs are recorded in real-time using a pair of 50 GHz balanced detectors connected to a 32 GHz oscilloscope. The recorded time-domain signals are then transferred to the Fourier domain via an FFT operation. The hybrid network introduces a $\pi /2$ radian phase delay between the I and Q components, as shown in the phase representation shown in Fig. 1a. It can be shown that the linear superposition of I-Q signals preceded by a $\pi /2$ phase rotation (i.e. multiplication by $i$) of the complex plane of one of the signals at the computation stage results in the realisation of the single-sided reconstruction of the complex optical field spectrum of the radiation being investigated. An inverse FT of this complex spectrum thus directly leads to the full-field time-domain representation of the signal being investigated (Fig. 1b).

The nonlinear spectrum is then calculated from the full-field information using the Zakharov-Shabat spectral approach highlighted in Eq. (2). Its solution renders the NFT decomposition of the pulse, giving the desired nonlinear spectrum. Figure 1c shows the flow diagram for the pulse processing, wherein field components from the time domain are subject to the direct NFT, transferring the problem to the NF spectral domain. Figure 1d displays the exemplary discrete NF spectra for the single laser pulse shown in Fig. 1b. The imaginary parts of the resulting eigenvalues are directly proportional to the intensity of the soliton and the real parts of eigenvalues, to the group velocity of each individual solitary mode.

### Normalisation considerations

An important step for arriving at the NFT spectrum is the normalisation of the input field, scaling the time and propagation distance. The relation between the NFT spectrum and the time-domain signal is highly nonlinear and significantly changes with the variation of the normalisation parameters. Thus, it is essential to ensure an understanding of how the NFT results where arrived at, and how they may be replicated. It is important for the application of the NFT analysis that the form of normalisation chosen is clearly defined at the start. As such then, a priori detailed knowledge of the system under investigation is not required, and in this notion, the NFT analysis takes the form of a pure signal processing approach.

Using experimental measurements, we thus demonstrate two ways by which the NFT can be used to analyse non-stationary laser dynamics. Firstly, we apply a universal $z$-independent field normalisation, then scrutinise the specific features of the resulting NF spectrum, using the information obtained to distinguish corresponding structures in time domain. Specifically, we normalise the laser field $Q(T,Z)$ or all values of $Z$ and $T$ by constant values of ${Q}_{{\mathrm{s}}},{T}_{{\mathrm{s}}}$ and ${Z}_{{\mathrm{s}}}$ leading to the change of variable $q(t,z)=Q(T,Z)/{Q}_{{\mathrm{s}}}$, where $t=T/{T}_{{\mathrm{s}}}$, $z=Z/{Z}_{{\mathrm{s}}}$. Note that the value of $Q(T,Z)$ is taken directly from our experiment, where real world units are used for measuring the physical quantities. This approach thus emulates what would happen to our pulse when the latter is launched into a lossless fibre span with coinciding characteristics, and is similar to that used in previous work^44^. Secondly, we show how an appropriately chosen normalisation for the NFT can be used to derive approximate equations governing the complex nonlinear intracavity dynamics. The variants of this approach are widespread for the description of dissipative solitons^59^. We emphasise that the second approach can be used to derive the approximate (underlying) model using the experimental results, contrary to the methods based on using a priori established models to describe observable properties.

### NFT of the laser radiation using z-independent normalisation

As the I-Q components of the laser radiation are monitored in real-time, we can analyse how the NF spectrum evolves over round trips applying the methodology of spatio-temporal dynamics.

**Fig. 2:**
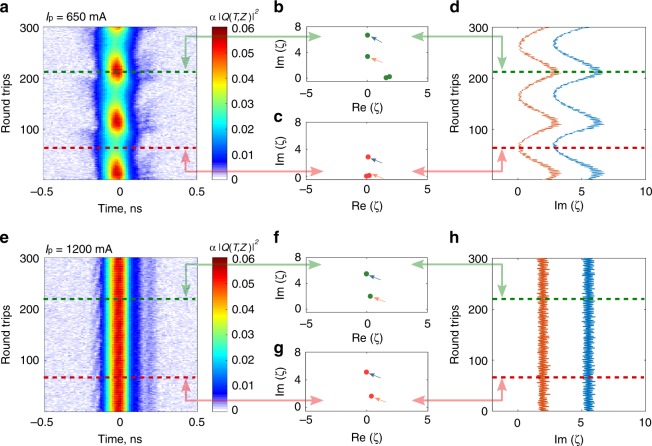
Real-time NFT data evolution obtained from the measurement of the fibre laser field. **a**, **e** Real-time spatio-temporal dynamics of laser evolution obtained from full-field measurements. **a** Corresponds to the pump current value ${I}_{\mathrm{p}}$ = 650 mA just above the lasing threshold ${I}_{{\mathrm{th}}}=600\; {\mathrm{mA}}$, while **e** refers to the case ${I}_{{\mathrm{p}}}$ = 1200 mA ($\gg\! {I}_{{\mathrm{th}}}$). **b**, **c**, **f**, **g** Instantaneous NFT discrete spectra obtained at the round trips marked by horizontal coloured lines in (**a**, **e)**. **d**, **h** Real-time evolution of the imaginary parts of two largest discrete eigenvalues indicated by arrows in (**a**, **e**).

Before the I and Q signals are superposed, they are segmented into contiguous round trip length windows ($\sim$62.85 ns), with the mode-locked pulses roughly centred within them. The FFTs of the windowed I and Q signals were then superposed to retrieve the full-field spectra. The spectral resolution is of the order of the mode spacing ($\sim$15 MHz), which is three orders of magnitude higher than that of the OSA ($\sim$0.02 nm) (see Supplementary note 1). The full-field spectral dynamics as obtained from the I-Q measurements are then subject to a row-wise Fourier transformation to arrive at spatio-temporal dynamics of Fig. 2. Figure 2 shows the dynamics of the fibre laser just above the laser threshold, Fig. 2a, where it is seen to be less stable, while well above threshold, Fig. 2e, it approaches a more stable emission regime.

To calculate the NF spectra from these real-time full-field dynamics, we adopt a universal z-independent normalisation strategy. Specifically, we normalise time to a time scale ${T}_{{\mathrm{s}}}= 0.1$ ns, which is approximately equal to the inverse of the signal bandwidth (containing 98% of the signal energy) averaged over many round trips. The amplitude is also normalised with a scale ${Q}_{{\mathrm{s}}}$, which is the squared root of the power of a Gaussian signal with a time-width equal to a time window containing $98 \%$ of the signal energy, amounting to ${Q}_{{\mathrm{s}}}^{2}=0.16$ W in our case.

As mentioned earlier, for the pure NLSE the eigenvalues remain constant over the propagation. However, as the normalisation applied here is z-independent, it is expected that the solitary mode content will change with variations of the laser pulse energy over round trips. Indeed, as seen in Figs. 2b and c, the discrete spectra are noticeably different for distinct $z$ values, corresponding to extremal excursions in the pulse dynamics. In Fig. 2d, h we depict the evolution of the imaginary parts for the highest eigenvalues with round-trip resolution. Figures 1d and 2b and c, thus, reveal that the analysed laser pulse has the structure with multiple embedded solitary NF modes and can be approximately represented as a nonlinear superposition of these modes (disregarding the dispersive components).

Eigenvalues with large imaginary parts have almost zero real parts, which indicates that these components of  the multi-soliton has a zero velocity in the reference frame of observation. Also, some eigenvalues have a non-zero real part, and a relatively small imaginary part, which is indicative of non-zero velocities of the corresponding time domain features in the reference frame.

The appearance of these features directly corresponds to the periodically appearing low intensity whisker-like signal components in the spatio-temporal picture, moving with the velocity different from that of the main laser pulse. At higher powers (Fig. 2e), the NF spectra remain constant over round-trips, and the eigenvalues become steady and stationary features, as seen in Fig. 2f, g. This one-to-one correspondence between the NF spectra and the emergent features in the spatio-temporal dynamics demonstrates the effectiveness of the NFT formalism in revealing the underlying solitary modes embedded into the laser pulse even though the system is not necessarily integrable^60^. Indeed, such an approach of using eigenvalue spectra has also been demonstrated by Ryzckowski et al.^44^, where several single-shot eigenvalue spectra for mode-locked laser pulses, qualitatively similar to Fig. 2b, c, f and g, were observed. The normalisation adopted therein was similar to z-independent normalisation convention presented here, and hence the interpretations of the dynamics by those authors were similar, including the association of low-lying eigenvalues with pulse pedestals.

**Fig. 3:**
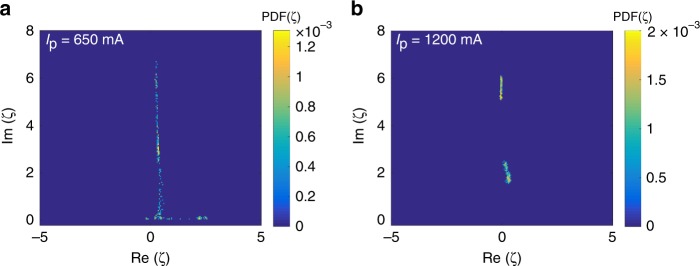
Eigenvalue distribution in the complex plane of spectral parameters—Distribution of the NFT discrete eigenvalues (depicted on a complex plane of nonlinear spectral parameter $\zeta$), corresponding to the operational regimes of Fig. 2a, e obtained for an ensemble of 1000 mode-locked pulses. The improved stability of the laser at the higher power is evident from the well-defined localised spots in the distribution of eigenvalues.

In addition to the above, the collective dynamics of such complex regimes that we have in our case can then be classified by a statistical interpretation of the eigenvalue excursions. The colour-map projections in Fig. 3 show the two-dimensional distribution of the eigenvalues on the complex plane for the two pump values. These distributions were obtained from the eigenvalue ensemble computed from 1000 consecutive pulse measurements. The instability exhibited by the laser at lower pump current powers is revealed as an unresolved picture containing a single elongated spot in Fig. 3a. The picture in Fig. 3a emerges due to the rapidly changing positions of individual embedded eigenvalues, with considerable deviation from their initial values. The existence of the periodically recurring low-lying eigenvalues can also be observed on that distribution. However, the lasing stabilisation at higher pump powers can be seen from the clustering and well resolved separation of the eigenvalues in Fig. 3b. From that picture it is then clear that at higher powers the lasing regime becomes stable, with the NFT revealing well-defined higher-order solitary modes content. The eigenvalue distributions can thus be used to define measures of structural, coherence, and stability characteristics of the laser, and to reveal the existence of intermittent dynamical features of the coherent pulse content, if any. In summary, the results presented herein highlight the potential of using a uniform z-independent normalisation to obtain real-time NFT spectra to reveal how the complex eigenvalues, i.e., the coherent content in the radiation, evolve over time.

### Deriving a laser model using z-dependent normalisation

The correspondence between the evolutionary behaviour of the signal and that of its nonlinear spectrum shows that the conventional reading of the nonlinear spectrum, i.e., a decomposition of the signal into its solitary and dispersive NF spectrum components, provides useful information about the particular dynamical regime. However, the NFT framework also makes it possible to get a quantitatively correct picture of the nonlinear pulse dynamics.

Let us take the laser output, $q(t,z)$, as a function of retarded time, $t$, and slow distance, $z$. We take the laser field at a discrete set of instances after each round trip, and assume that those instances are given by the continuous variable $z$ with the one round trip increment. Our time $t$ is measured in the frame co-moving with the group velocity of the envelope $q(z,t)$. The signal, $q$, evolves according to the equation,

5 $$i\frac{\partial }{\partial z}q(t,z)={\frak{L}}(q)q.$$

If the r.h.s. of the above equation is linear, the FT can be used to find a solution to Eq. (5) at a given distance $z$. A similar procedure can be used in the case of nonlinear ${\frak{L}}$, when the system is integrable or nearly integrable. In the latter case we assume that it still keeps some important properties of an integrable system.^61^ Since some particular integrals of motion may not be conserved given the system is perturbed, the important information about the behaviour of the perturbed system can be inferred by our inspecting the alternations of those motion integrals as the waveform evolves. When the perturbation cannot be considered as a small one, but is periodic, the other perturbation approach based on averaging can be used^62^.

In fibre lasers, quite often the dissipative (gain, loss) and conservative (dispersion, Kerr nonlinearity) effects are effectively separated. For instance, combined effects of gain and loss (both being saturable) are responsible for growth of the optical field from initial noise and stabilisation of energy at a certain level. In turn, fibre dispersion and Kerr nonlinearity are responsible for pulse shaping. Therefore, often, coherent structures observed in fibre lasers can be explained by the solutions of the pure NLSE (fundamental and higher-order solitons, Peregrine soliton, breathers, and so on). This makes NLSE an inherent part of the mathematical description of optical field dynamics in fibre lasers as well. Based on the observation of the correspondence between the nonlinear spectrum evolution and the signal’s behaviour from round trip to round trip, we assume that the operator in Eq. (5) contains the pure NLSE part (see e.g. ref. ^59^). Then, taking into account the character of eigenvalues’ variations, we assume that the pulse dynamics can be approximately modelled by the perturbed NLSE, which we write down here in the general form

6 $$i\frac{\partial q}{\partial z}+\frac{{\partial }^{2}q}{\partial {t}^{2}}+2q| q{| }^{2}=G[z;\ q,\ \ldots ],$$

where $G[\cdot ]$ is some periodically recurring function in $z$ with possibly large local variation. The periodicity of $G[z;\ldots ]$ means that under some conditions on the interplay between the typical nonlinear distance and the period of $G[z;\ldots ]$, we can use the theory from e.g. ref. ^62^ to derive a path-averaged NLSE describing the soliton propagation in the leading approximation. Thanks to a rich body of research on NLSE perturbation methods, it is possible to start from the evolution of the nonlinear spectrum and estimate some perturbing terms in the model, i.e., $G[z;q,\ldots ]$.

To take advantage of such methods dealing with perturbed NLSE-type solitons (*sech-* pulse profiles), we now choose a particular set of normalisations for time, $t$, and amplitude, $q$, where the resulting nonlinear spectrum would contain a minimum amount of continuous spectrum and the normalised solution can be with a high accuracy considered as a signal containing only the solitary NF modes. Such a choice means that we effectively project the dynamics of our system onto the solitary eigenmodes of NLSE operator. In order to arrive at this kind of normalisation, we calculated the nonlinear spectrum of each round trip for various values of time and amplitude scales. Having obtained the continuous and discrete spectra, the energy content of these spectra, ${E}_{{\mathrm{c}}}$ and ${E}_{{\mathrm{d}}}$, are then numerically calculated for each $z$ using Eq. (15) from the Supplementary Material. Then, again at each $z$, that set of time and amplitude scale, which lead to the minimum value of continuous spectrum contribution, is selected as the normalisation parameters. In such a way, the normalisation becomes signal-dependent, and the same procedure is used for each value of $z$. This specific $z$- and signal-dependent normalisation effectively projects the dynamics of our system onto the evolution of NF solitary modes. We emphasise that such an approach can be realised only by means of the NFT signal processing method.

**Fig. 4:**
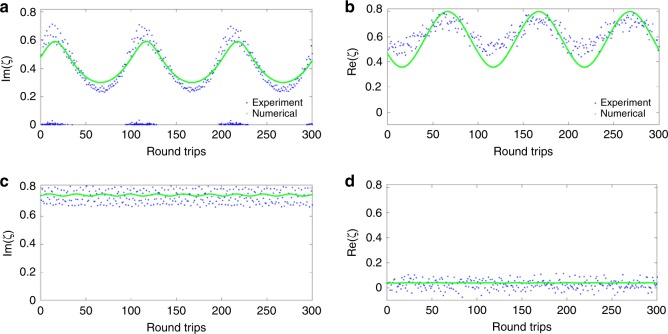
Collective coordinate (CC) analysis of laser radiation. **a** The imaginary part, and **b** the real part of the discrete spectrum of the output of the laser when ${I}_{\mathrm{p}}=650$ mA where the parameters of Eq. (8) are $\delta =-0.2$, $a=0.0619$, and $K=0.0196$. **c** The imaginary part, and **d**. the real part of the discrete spectrum of the output of the laser when ${I}_{\mathrm{p}}=1200$ mA where the parameters of Eq. (8) are $\delta =-0.3$, $a=0.1$, and $K=0.2$. These parameters obtained through a CC analysis of the laser output, can be used to arrive at a channel model for the laser system under study.

Next, we computed the NFT spectrum corresponding to the sequence of effective normalised profiles from above. Note that this effective projection approach is new and has not been applied to dissipative systems. From this analysis, we find that for all values of $z$ the NF spectrum of the resulting projected system contained just one discrete eigenvalue, see Fig. 4. Now, making use of Eq. (6), we can employ a relatively simple approach provided by a variant of a variational approach specifically applicable for the study of localised excitation, the collective coordinate formalism^63,64^. Utilising such a technique we aim at arriving at a simplified model that describes the experimental results. In our case, we matched the dynamics of eigenvalues, Fig. 4b, with that corresponding to the trial soliton-type ansatz^63^:

7 $$q(t,z)=2i\eta (z)\,{\mathrm{sech}}\,\left[2\eta (z)\left(t-\sigma (z)\right)\right]{e}^{-i\left(2\xi (z)t+\phi (z)\right)}.$$

With the help of that matching, we then derived an approximate model to describe the pulse evolution using the dynamics of four soliton parameters from Eq. (7) taken as collective coordinates: $\eta$, $\sigma$, $\xi$ and $\phi$. This model is a perturbed NLSE where $G[z;q, \ldots ]$ in Eq. (6) is:

8 $$G[z;\; q, \ldots ]={E}_{{\mathrm{in}}}-i\beta q(t,z)-\delta q(t,z),$$

with ${E}_{{\mathrm{in}}}=a{e}^{iKt}$ and real parameters $a$, $K$, $\beta$ and $\delta$^63^. Now we apply the technique known from the analysis of discrete breathers^65^ and find the matching parameters of our effective perturbation term (8) according to the observed evolution of the experimental samples. The numerically obtained dynamics shown in Fig. 4 is obtained for pump currents of ${I}_{\mathrm{p}}=650$ and $1200$ mA. The real and imaginary part of the single eigenvalue, $\xi$ and $\eta$ in Eq. (7) respectively, are depicted for both the calculated nonlinear spectrum from the experimental samples and for the one that was obtained from numerically solving the collective coordinate system of equations^63^. Equation (8) can be viewed as describing the effective perturbations that our systemʼs projection onto the solitary mode experiences: this mode is affected by the effective external pumping, attenuation, and central-frequency shift. However, these effects must not be attributed to the initial dynamical systems, as they pertain specifically to the projection of our initial system onto the solitary NF mode. Note that in Eq. (8), $\beta$ effectively plays the role of a damping factor and since the evolution of the observed experimental samples do not show any decay, we set $\beta =0$ from the beginning of our procedure.

The approach depicted in the current section provides a universal normalisation scheme and the respective attached tool to derive the system governing equation and its parameters by matching the results of the NFT analysis with the experimental samples. Of course, the eigenvalues from Fig. 4 are physically different quantities compared to those of the NF spectrum depicted in Figs. 2 and 3. This difference shows that the NFT-based system analysis can be applied in a number of different ways and tailored for studying different aspects of the system’s dynamics, and shows the considerable difference in the way the nonlinear spectrum is to be interpreted if the underlying system is non-integrable.

## Discussion

The study of complexity in physical systems introduced by nonlinearities is a thriving interdisciplinary area of research that benefits extensively from a cross-pollination of ideas between the different fields. The universality of the underlying mathematical models particularly enables effective transfer of knowledge and techniques across disciplines. In this paper, we have demonstrated the application of NFT based on the Zakharov-Shabat spectral problem for the analysis of fibre laser radiation. This approach offers a natural framework for the description of localised (in time) coherent features embedded in the dispersive waves. The discrete eigenvalue spectrum is extremely sparse, and yet encompasses essential information about the dynamics being investigated. We have shown in our paper that the eigenvalues map very well the features observed in the spatio-temporal dynamics, making the interpretation of the eigenvalue spectrum simple. For the mode-locked laser used here as a test-bed, we have shown how NFT signal processing based on the Zakharov-Shabat spectral problem can help in the understanding of the underlying dynamics in terms of a soliton decomposition of the pulse features. The real-time measurement approach allows one to track appearance of coherent, solitonic features with round-trip resolution, which can be directly attributed to intensity domain features spotted in the spatio-temporal dynamics. Acquisition of large ensembles enable statistical studies of the dynamics, which can further help classify the operational regime on a multi-dimensional parameter space. Furthermore, we have also shown how existing mathematical models for lasers can be suitably adopted to explain observations, and hence facilitate the crucial cross-pollination of ideas mentioned above. While for the current laser the energy in the continuous spectrum is much lower in comparison to the discrete eigenvalue spectrum, in systems exhibiting co-existence of solitary modes and dispersive waves, the continuous spectrum can be used to reveal energy exchanges between them specially in the pulse forming and decaying stages^66^.

Here, the coherent homodyne detection configuration has been used for recovering the full-field information of the laser output for reconstructing the NFT. This methodology is complementary to the digital temporal hologram approach adopted by Tikan et al.^54^, and the time-lens and dispersive FT based methodology by Ryczkowski et al.^44^ that used iterative algorithms providing full-field recovery over THz-bandwidths. This high-bandwidth advantage cannot be discounted, especially when considering laser systems. However, time-lens configurations are complex, and rely on synchronisation between the test and the time-lens pump lasers, and this indeed proved to be challenging when investigating transient, non-steady regimes of the laser. In this regard, the coherent detection methodology used here is indeed complementary. It utilizes an industry-tested configuration that has a compact form factor, which while currently limited in bandwidth, allows for amplification of weak signals, and also offers a much higher spectral resolution, which can potentially reveal spectral dynamics down to the level of individual lasing modes. Phase information is determined deterministically using quadrature phase detection. Importantly, no synchronisation arrangement is required, and measurements can be made continuously without risking pulse dropouts due to walk-off effects. The record length is limited by the oscilloscope memory, and can reach several tens of thousands of round-trips for typical metre-length fibre lasers. Furthermore, in comparison to DFT based methodologies, it can also be used in regimes where mode-locked and dispersive features co-exist. While the calculation of the NFT based on the Zakharov-Shabat spectral problem is numerically expensive, its FFT-variations can be exploited for code speed-up. Together with the spatio-temporal methodology, the NFT can help reveal the evolution of such coherent features in real-time. In particular, it can help capture highly transient events and the events leading to them, as in the generation of rogue waves. The experimental methodology can also be expanded to account for polarisation diversity of the radiation in the study of Manakov-like systems. The demonstrated techniques are not limited to fibre lasers, and can be extended to the study of other lasers^50^, optical signals^67^ and partially coherent light sources in general. Utilising NFT jointly with other well-known techniques, like collective coordinates, can help modelling some of these light sources. The full-field information made available by the methodology, together with the information about the nonlinear content provided by the NFT can help understand better the underlying nonlinear dynamics in a wide range systems, and can be used to establish a *lingua franca* for communicating ideas across disciplines.

## Methods

### Nonlinear spectrum computations

To calculate the nonlinear spectrum of the output of the laser, complex full-field samples measured using the IQ methodology at 12.5 ps time intervals are used. The normalisation is done using the specific parameters sets as explained in the main text. The average signal power for each patch of signal for hundreds of round trips is fixed to the power measured using a power meter and by considering the 90:10 coupler which couples out a portion of the output to a single mode fibre (SMF) to the measuring device. The NFT calculations are carried out with the number of samples that vary from 500 to 5000 depending on the time window as explained above. The signal consists of a coherent Gaussian-like pulse with long zero wings which makes the vanishing boundary assumption valid to assign discrete and continuous spectrum as the nonlinear spectrum (see ref. ^45^ for details). While the continuous spectrum turns out to constitute a small fraction of the nonlinear spectrum in our experimental samples, hence, ignored, it can carry some information particularly about the transition of energy in the nonlinear domain when considerable^66^. However, negligible continuous spectrum helps implement several techniques which are mainly designed to study the dynamics of solitons.

To compute the NF spectrum we consider the discretized variant of Zakharov-Shabat problem (2). The fast Ablowitz-Ladik algorithm^68,69^ for the computation of NFT quantities is used in our study to find the nonlinear spectrum. This algorithm has been shown to be one of the most efficient ones in which the scattering parameters, $a(\zeta )$ and $b(\zeta )$ (see Eq. (4) and Supplementary Note 2 for more details), are calculated by numerically solving the specifically-discretised Zakharov-Shabat system (2). For the recent review and comparison of the NFT computation methods see^70^. We consider the ring laser as an SMF fibre with discrete optical components such as polarisation rotation plates, linear filter and active fibre as an amplifier. As explained in previous sections, this model implies that the integrable Schrödinger equation, to which the additional optical components play the role of perturbing terms, is the one describing an SMF.

## Supplementary information

Supplementary Information

## Data Availability

The data that support the findings of this study are available from the corresponding author upon reasonable request.

## Code availability

Code for implementing the NFT algorithm is available as an open source resource^69^. Code for implementing the spatio-temporal representations is available from the corresponding author upon reasonable request.
