# Author Correction: Analysis of laser radiation using the Nonlinear Fourier transform

**DOI:** 10.1038/s41467-020-18971-y

**Published:** 2020-10-02

**Authors:** Srikanth Sugavanam, Morteza Kamalian Kopae, Junsong Peng, Jaroslaw E. Prilepsky, Sergei K. Turitsyn

**Affiliations:** 1 Aston Institute of Photonic Technologies, Aston University, Aston Triangle, Birmingham, B4 7ET UK; 2 State Key Laboratory of Precision Spectroscopy, East China Normal University, Shanghai, 200062 China; 3 Aston-Novosibirsk International Centre for Photonics, Novosibirsk State University, Novosibirsk, 630090 Russia

**Keywords:** Fibre optics and optical communications, Fibre lasers, Mode-locked lasers, Nonlinear optics

Correction to: *Nature Communications* 10.1038/s41467-019-13265-4, published online 11 December 2019.

The original version of this Article omitted the following from the Acknowledgements:

This project has received funding from the H2020 MSCA-RISE-HALT under grant agreement No. 823937.

This has now been corrected in both the PDF and HTML versions of the Article.

