# Supplementary material for: Analysis of laser radiation using the Nonlinear Fourier transform
Source: Nat Commun. 2019 Dec 11;10:5663. doi: 10.1038/s41467-019-13265-4 (PMC6906527; doi:10.1038/s41467-019-13265-4)
Supplement: Supplementary file 1 — Supplementary Information [file 41467_2019_13265_MOESM1_ESM.pdf]

Supplementary material - Analysis of Laser Radiation Using the Nonlinear Fourier Transform

Srikanth Sugavanam[1,+], Morteza Kamalian Kopae[1,+], Junsong Peng[2], Jaroslaw E. Prilepsky[1], and Sergei K. Turitsyn[1,3,*]

[1]Aston Institute of Photonic Technologies, Aston University, Aston Triangle, Birmingham B4 7ET, United Kingdom
[2]State Key Laboratory of Precision Spectroscopy, East China Normal University, Shanghai 200062, China
[3]Aston-Novosibirsk International Centre for Photonics, Novosibirsk State Univeristy, Novosibirsk, 630090, Russia
[*]s.k.turitsyn@aston.ac.uk
[+]these authors contributed equally to this work

Supplementary Note 1 - Coherent homodyne detection for full-field characterization

A comprehensive understanding of the underlying dynamics exhibited by radiation is possible if information about its amplitude (or alternatively, intensity) and phase evolution is available. However, conventional photodetectors generate photocurrents proportional only to the instantaneous intensity, and all information about carrier phase is lost. Further, the limited bandwidth of state of the art electronic devices pose a limitation on the direct detection of the carrier phase to optical frequencies of up to a few tens of gigahertz. Mixing techniques help bring optical frequencies from the terahertz range down to frequencies accessible by existing electronic oscilloscopes and analyzers. Yet, recovery of phase remains challenging. For instance, heterodyne detection is widely used for the characterization of narrow linewidth radiation. In principle, the method is phase sensitive, and indeed under slowly varying envelope approximations, the underlying phase evolution can be recovered[1]. However, the method also retains phase ambiguity. The coherent homodyne detection methodology employed in the current paper helps resolve this phase ambiguity, and helps recover information about both intensity and phase, i.e. full-field information.

In homodyne detection, two copies of the signal are generated which are shifted by a phase difference of $\pi/2$. The phase shift is facilitated by the 90^0 hybrid. The frequency of the narrow linewidth local oscillator laser is chosen to be close to that of the lasing (hence the homodyne connotation). The signal in-phase with the original signal and the one that is quadrature are separately mixed with the local oscillator and measured using the digital storage oscilloscope (see Fig. 1 of the main article for the experimental configuration). The following is a very simple explanation of how a straightforward addition of the complex valued Fourier transforms of the I and Q signals yield the full-field spectrum.

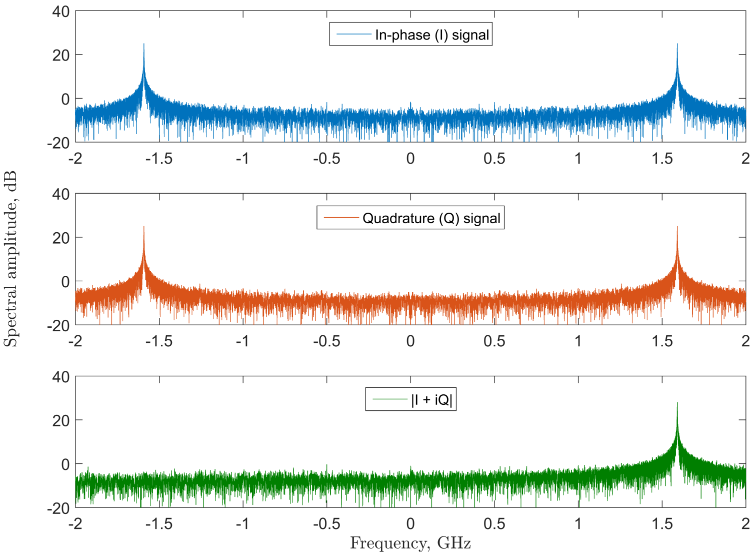

Supplementary Figure 1: Recovering the full-field spectrum - Fourier transforms of experimentally measured in-phase I (red) and quadrature Q (orange) signals for a narrow linewidth laser. A multiplication of the Q-transform by i, followed by its linear superposition with the I-transform yields the complex-valued singled sided spectrum of the narrow linewidth laser.

Say the signal of interest arrives from a monochromatic laser of optical frequency ν_L. Let the optical frequency of the local oscillator ν_{LO} be slightly different from it, so that the detected homodyne signals have an angular frequency $\omega = 2\pi(\nu_L - \nu_{LO})$. The corresponding in-phase and quadrature components can be written as

$$I_{\text{in}}(t) = \cos(\omega t) \quad (1)$$

$$= \frac{1}{2}[\exp(i\omega t) + c.c.], \quad (2)$$

$$I_{\text{quad}}(t) = \cos(\omega t + \pi/2) \quad (3)$$

$$= \frac{1}{2}[\exp(i\omega t).\exp(i\pi/2) + c.c.]. \quad (4)$$

ω can be referred to as the mixed-down frequency. A real-world signal may also contain additional intensity and phase terms; here they have been dropped for clarity. The balanced detection scheme removes contribution from direct terms[2]. From

equations 2 and 4, it is evident that the Fourier transform of a real-world signal will yield both positive and negative frequencies, with the latter corresponding to the contribution of the complex conjugate terms ($c.c.$).

Then, the superposition of the I and Q signals, after the Q signal is phase-rotated by $\pi/2$, facilitated by a multiplication by $\exp(i\pi/2)$, i.e. i yields the single sided spectrum as follows -

$$I_{\text{in}}(t) + iI_{\text{quad}}(t) = \frac{1}{2}[\exp(i\omega t) + \exp(i\omega t - i\pi/2).\exp(i\pi/2) \tag{5}$$

$$+ \exp(-i\omega t) + \exp(-i\omega t + i\pi/2).\exp(i\pi/2)], \tag{6}$$

$$= \exp(i\omega t). \tag{7}$$

The resulting summation yields a single side spectral representation of the signal, which consists of both intensity and phase information. Similarly, it can be shown how coherent homodyne detection allows for observation of optical bandwidths that are larger than the effective electrical bandwidth. Say the radiation under study consists of two frequency components $\omega_s + \Delta\omega$ and $\omega_s - \Delta\omega$, where ω_s is the optical frequency, and the offset frequency $\Delta\omega$ does not exceed the electrical bandwidth of the measurement configuration. In this case, if the optical frequency of the local oscillator is also chosen to be ω_s, the in-phase and quadrature components can be written as

$$I_{\text{in}}(t) = \frac{1}{2}[\exp(-i\Delta\omega t) + \exp(i\Delta\omega t) + c.c.], \tag{8}$$

$$I_{\text{quad}}(t) = \frac{1}{2}[\exp(-i\Delta\omega t).\exp(i\pi/2) + \exp(i\Delta\omega t).\exp(i\pi/2) + c.c.], \tag{9}$$

so that their linear superposition yields

$$I_{\text{in}}(t) + iI_{\text{quad}}(t) = \exp(-i\Delta\omega t) + \exp(i\Delta\omega t). \tag{10}$$

If we envision now that $\Delta\omega$ has a value close to the electrical bandwidth of the measurement configuration, equation 10 shows that the total observable optical bandwidth is $2\Delta\omega$, i.e. double the electrical bandwidth. The doubling can thus be attributed to the contribution of the negative frequencies. This also shows why it is beneficial to choose the optical frequency of the local oscillator to be close to the central frequency of the test radiation. Note however that the temporal domain resolution remains unaffected, as this is related to the frequency domain resolution via the transform relation.

Round-trip time resolved, full-field spectro-temporal dynamics

As mentioned in the main article, the real-time I/Q signals were subjected to the spatio-temporal methodology[1,3] for arriving a round-trip time resolved representation of the laser's spectro-temporal dynamics. To reiterate, the real-time I and Q signals obtained using the digital storage oscilloscope were first segmented into contiguous windows of length equivalent to the round-trip time of the laser. These segments were stacked atop one another to arrive a two-dimensional matrix representation of the I and Q signals. An FFT operation was then performed row-wise on them. Then, following the procedure highlighted in Supplementary Note 1 above, the Q signal matrix was phase rotated by multiplying each element of it with $\exp(i\pi/2)$, and added to the I matrix to yield the round-trip time resolved, full-field spectro-temporal evolution of the laser radiation.

Supplementary Figure 2 shows the two-dimensional representation of the spectral power (i.e. square of the absolute value of the FFT) for the two different pump powers as discussed in the main text, where the rows correspond to consecutive round trips. To maximize spectral resolution, no additional windowing functions were used. Any errors introduced due to this can be considered minimal as the background radiation is close to zero. The averaged characteristics are in good agreement with the spectra measured using a conventional optical spectrum analyzer (Supplementary Figure 3). The observed dips in the intensity arise from floor-noise removal (for zero-signal input), to mitigate the effect of instrumental bias and noise. For the real-time spectra, the spectral resolution is of the order of the mode spacing ($\sim$15 MHz), which is three orders of magnitude higher than that of the OSA ($\sim$0.02 nm). The manifestation of the higher resolution is clearly seen in the resolution of the continuous wave peak in Supplementary Figures 2b, d, which is integrated and smoothed out by the OSA (Supplementary Figure 3, dotted curve at higher power). This is a clear demonstration of the advantage and necessity of using high resolution spectral measurements when studying and interpreting laser dynamics.

As the single-sided spectral reconstruction yields full-field information, information about spectral phase can also be reconstructed for each pulse. The grey curves of Supplementary Figures 2c, d show the spectral phase across a given pulse, relative to that at the peak spectral frequency. For the higher power case, the phase was measured relative to that of the continuous wave peak. The orange curve is the average phase obtained over 1000 consecutive pulses. These dynamics are subjected to a row-wise inverse FFT transformation to arrive at the full field dynamics shown in Fig. 2 of the main article.

The full-field spectral dynamics as obtained from the I-Q measurements were subject to a row-wise Fourier transformation to arrive at the corresponding pulse characteristics in Fig. 1 of the main text, and the spatio-temporal dynamics of Supplementary

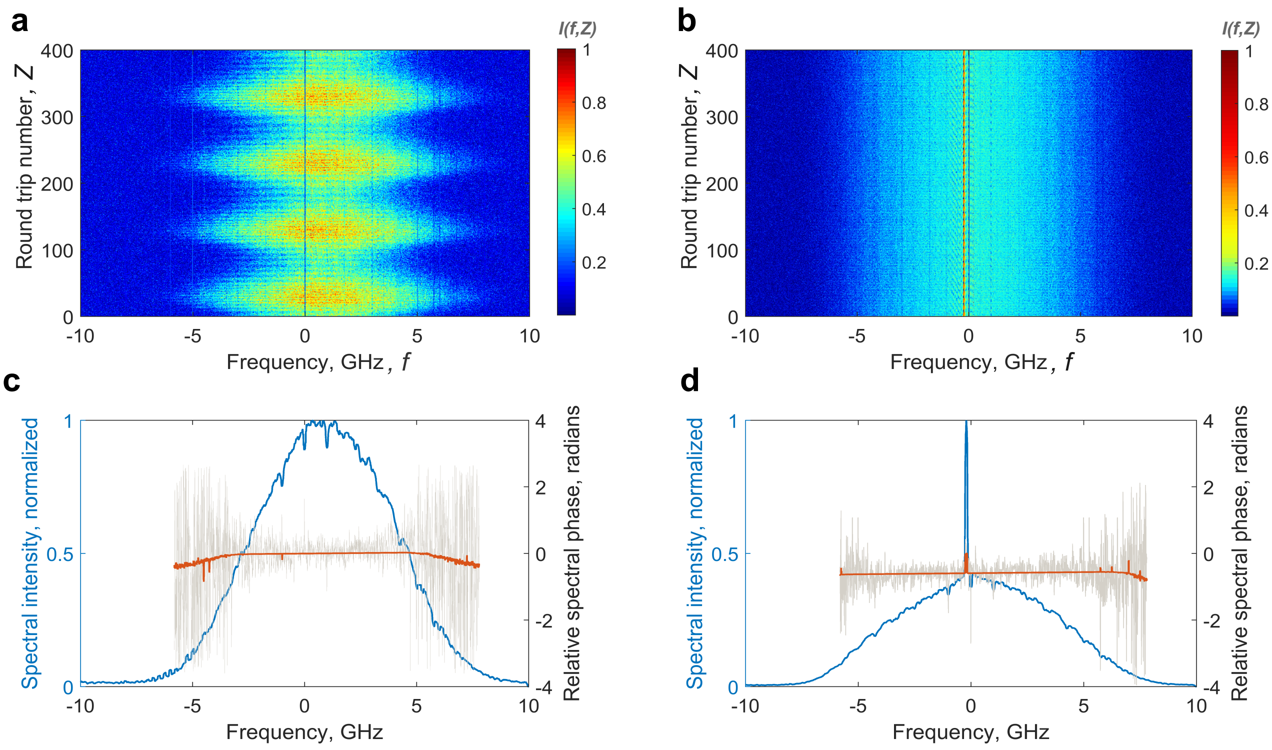

Supplementary Figure 2: Real-time spectral dynamics - a,b. Spectrally resolved intensity evolution (normalized), resolved over round-trips at $I_\mathbf{p} = 650$ mA and $I_\mathbf{p} = 1200$ mA respectively. **c,d.** Averaged spectral intensity, (blue) and spectral phase (orange) for the corresponding pump powers. The grey curves indicate a round-trip resolved instance of the spectral phase.

Figures 2a,d. The instantaneous frequency chirp was estimated as the first-order difference of the temporal phase across the pulse. The value of the instantaneous frequency at the centre of the pulse was taken to be zero. A linear fit of the instantaneous frequency chirp gave a value of its slope as 25 nm/GHz for both lower and higher powers (similar to that in the spectral domain).

Supplementary Note 2 - Nonlinear Fourier transform computation

In this section of the supplementary we define some terms and concepts related to the inverse scattering or nonlinear Fourier transform. As mentioned earlier, IST is a mathematical method for solving nonlinear differential equations in a way similar to applying Fourier transform to linear evolution equations[4]. To put it in a more rigorous term, for some nonlinear partial differential equations, NLSE being one of them, one can find a pair of ordinary linear differential equations (LODE), represented by the so-called Lax pair operators $\hat{M}, \hat{L}$, with the following properties[5]:

- M and L in general depend on, $q(t,z)$, the fast decaying solution to the NPDE,
- For a solution, $\Phi = [\phi_1, \phi_2]^T$, to both $\hat{L}\Phi = \zeta\Phi$ and $\Phi_z = \hat{M}\Phi$, the compatibility condition $\Phi_{tz} = \Phi_{zt}$ is equivalent to $q(t,z)$ satisfying the nonlinear partial differential equation,
- for such $q(t,z)$, $\zeta_z = 0$.

For the particular case of NLSE, the equation for $\hat{L}$ is shown to be[6]:

$$\hat{L}\Phi \equiv \begin{bmatrix} i\partial_t & q(t,z) \\ -q^*(t,z) & -i\partial_t \end{bmatrix} \Phi = \zeta\Phi. \tag{11}$$

Solving Eq. (11) subject to the following "initial condition":

$$\lim_{t\to-\infty} \Phi = \begin{bmatrix} e^{-i\zeta t} \\ 0 \end{bmatrix}, \tag{12}$$

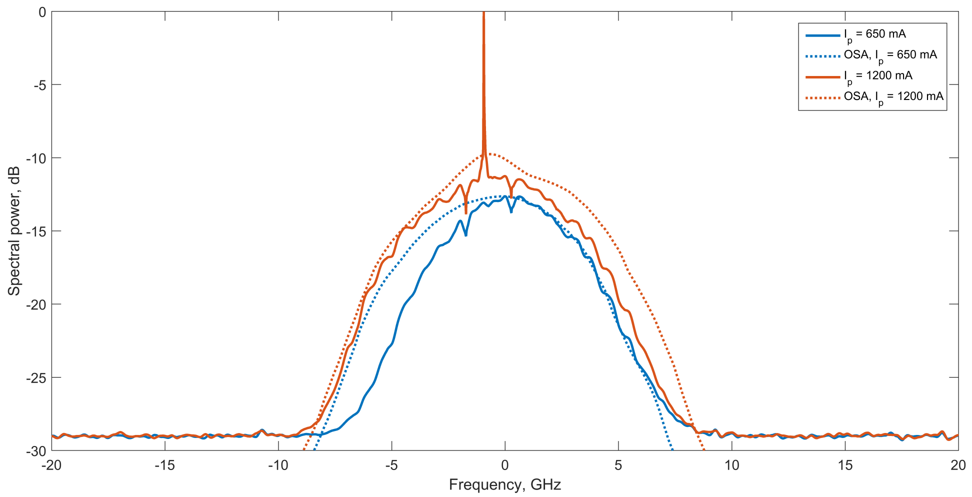

Supplementary Figure 3: Comparison of the spectra measured using a conventional optical spectrum analyzer (dotted curves) and those retrieved from the homodyne measurements (solid curves).

gives the scattering parameters determining the behaviour of Φ at $t \to \infty$:

$$a(\zeta) = \lim_{t\to+\infty} e^{i\zeta t}\phi_1, \quad b(\zeta) = \lim_{t\to+\infty} e^{-i\zeta t}\phi_2. \tag{13}$$

The scattering coefficients $a(\zeta)$ and $b(\zeta)$ constitute the essence of the NFT pulse decomposition. Using the scattering functions, $a(\zeta)$ and $b(\zeta)$, the nonlinear spectrum consisting of the continuous, $r(\zeta)$, and discrete specral amplitudes (norming constants), c_n, are defined as

$$\begin{aligned} r(\zeta) &= \frac{b(\zeta)}{a(\zeta)}, \quad \zeta \in \mathbb{R}, \\ c_n &= \frac{b(\zeta_n)}{a_\zeta(\zeta_n)}, \quad \zeta_n \in \mathbb{C} \text{ is a simple root of } a(\zeta), \end{aligned} \tag{14}$$

are calculated. The forward transform operation (finding the nonlinear spectrum from $q(t,z)$) for NLSE based systems can be obtained using the approach put forward by Ablowitz, Newell, Kaup and Segur – now known eponymously as the AKNS method – which is a generalized Lax methodology. The inverse transform operation (finding $q(t,z)$ from the nonlinear spectrum) was shown to be possible by Gel'fand Marchenko and Levitan, via a solution of a linear integro-differential equation. Here the nature of the solution is again reminiscent of the Fourier Transform operation, where the simpler Fourier exponential kernel is replaced by a more complex function.

For an equation solvable with this method, hence, integrable, there are infinite number of conserved quantities which, in essence, can be used to find a solution. These constants of motion can further be used to unravel the dynamics of the system. The conserved quantity we intend to use in this work is the energy of signal which is equal to the sum of the energy in the continuous and discrete spectrum, E_c and E_d respectively, defined below

$$E_c = -\frac{1}{\pi}\int_{-\infty}^{\infty} \log|a(\xi)|^2 d\xi = \frac{1}{\pi}\int_{-\infty}^{\infty} \log(1+|r(\xi)|^2), \qquad E_d = 4\sum_{i=1}^{N} \Im\zeta_i, \qquad E_t = \int_{-\infty}^{\infty} |q(r)|^2 dt, \tag{15}$$

where N is the total number of discrete eigenvalues (solitary modes). Although laser is not fully described by an integrable nonlinear system, the tangible physical meaning of the eigenvalues can be useful to interpret the underlying nonlinear dynamics. To this aim, the data emerging from the NFT analysis, such as the E_t, can be useful in the energy partition formula, Eq. (15). As mentioned before, the NFT decomposition preserves the energy which can be simultaneously defined using the time-domain, E_t, and the NF domain quantities from the continuous and discrete parts of the NF spectrum, E_c and E_d, correspondingly. Supplementary Figure 4 shows these energies calculated for each round trip for two different input pump powers. It is seen that the generated pulse can be treated as a signal with mostly solitonic components. Fluctuations in E_d follow exactly the variation of power in the time domain while the energy in the continuous spectrum remains almost unchanged. This variation is considerable in the less stable regime of near threshold excitation (pump current of 650 mA) while for the high power case

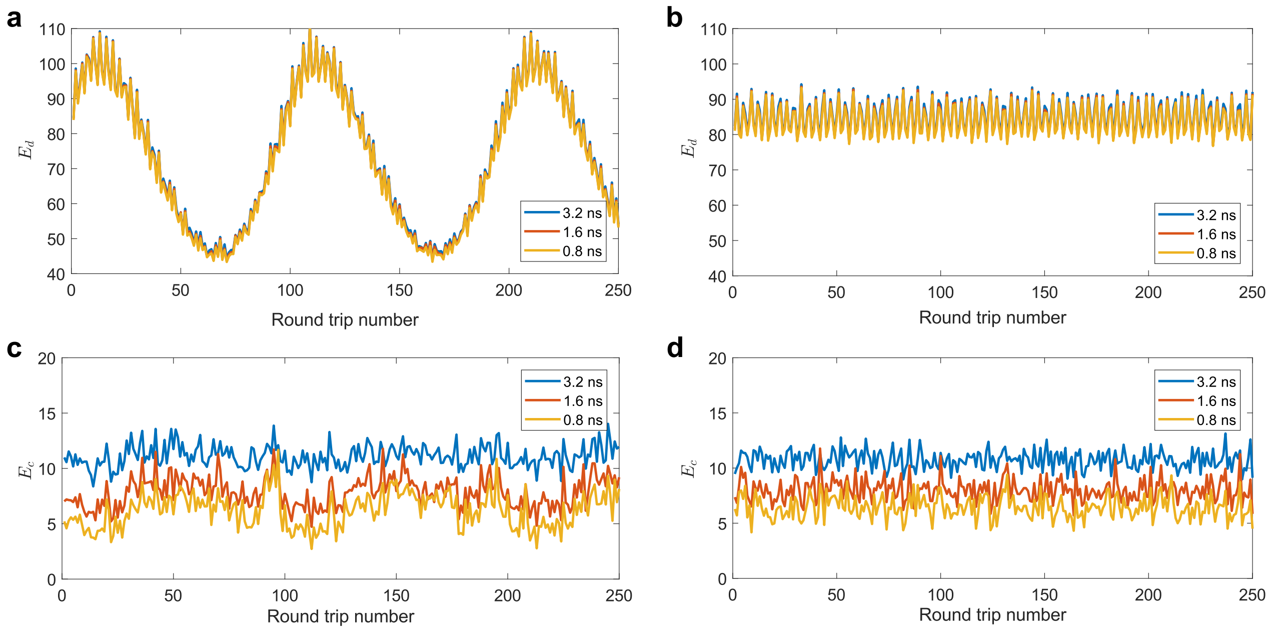

Supplementary Figure 4: Energy content in the discrete (a_1) and continuous (a_2) spectra of the laser output with $I_p \approx I_{th}$ and the discrete (b_1) and continuous (b_2) spectra of the laser output with $I_p >> I_{th}$ showing that the continuous spectrum constitutes a small fraction of the NS. It is also evident from these figures that changing the processing windows does not change the contents of the NS significantly.

(pump current of 1200 mA) it is merely a small perturbation. An interesting additional feature to look at in the NFT laser analysis is that the impact of the signal window in which the NFT calculations are carried out, on the overall NFT spectrum. Due to the effect of the saturable absorber which suppresses the low-power part of the signal, the wings of the pulse contain a small amount of energy especially when it comes to the discrete spectrum: changing the processing window has a negligible impact on E_d while the additional energy mainly contributes to the value of non-coherent content energy E_c, the dispersive part of the pulse, Supplementary Figure 4. This, in fact, justifies our choice of using vanishing boundary NFT for this particular type of laser and output and shows that the continuous spectrum is mainly related to the background noise.

A perturbation approach to analyse the evolution of the DS

As mentioned in the main text, taking into account the character of eigenvalues' variations, we assume that the pulse dynamics can be approximately modelled by the perturbed NLSE,

$$iq_z + q_{tt} + 2|q|^2 q = G[q,z,t], \tag{16}$$

where $G[q,z,t]$ is the perturbation, and the normalisation is explained in the main text, see subsection "Case 2". We assume a particular form for this perturbation as:

$$G[q,z,t] = E_{in} - i\beta q - \delta q. \tag{17}$$

We adopt a collective coordinate theory to study the dynamics of the system of Eq.(16). In such a theory, we start with an exact solution to the un-perturbed ($G[q,z,t] = 0$) equation and assume that during the propagation, the nature of the solution changes only in a way that it remains the same type of solution but with evolved parameters, called the collective coordinates. The number and kind of the collective coordinates can be different for different applications. Following[7], the 1-soliton solution to the non-perturbed (16) as below is considered

$$q(t,z) = 2i\eta \operatorname{sech}[2\eta(t-T)]\, e^{-i(2\xi t+\phi)}. \tag{18}$$

In the NFT paradigm, where the eigenvalue of the related ZS system is ζ and the spectral amplitude at this eigenvalue c, we have

$$\xi = \Re\zeta, \quad \eta = \Im\zeta, \quad \phi = -\arg c - \frac{\pi}{2}, \quad \zeta = \frac{\log\frac{|c|}{2\eta}}{2\eta}, \tag{19}$$

with the evolution in the non-perturbed NLSE[8]:

$$T(z) = T(0) - 4\xi z, \quad \phi(z) = \phi(0) + 4(\xi^2 - \eta^2)z, \tag{20}$$

Choosing these four collective coordinates, T, η, ξ, and ϕ and assuming the solution at each z to be of the form (18) and as the ansatz yields four entangled differential equations as follows:

$$\begin{aligned}
\dot{\eta} &= -2\beta\eta - a\frac{\pi}{2}\mathrm{sech}A\cos B,\\
\dot{\xi} &= aA\mathrm{sech}A\cos B,\\
\dot{T} &= -4T + \frac{a\pi^2}{8\eta^2}\mathrm{sech}A\tanh A\sin B,\\
\dot{\phi} &= -2T\dot{\xi} + 4(\xi^2 - \eta^2) - \delta + \frac{a\pi^2 A}{2\eta}\mathrm{sech}A\tanh A\sin B,
\end{aligned}$$

with

$$A = \frac{\pi}{4}\frac{K+2\xi}{\eta}, \qquad B = \phi + (K+2\xi)T.$$

Our goal is to starting from the discrete spectrum coming from the experimental results, and by using a set of fix normalisation constants, find the model parameters, β, a, K and δ which best describe the evolution of ζ in z (roundtrip) direction. Slightly changing the parameters of the model (of course, not always and not all of them) leads to a modest variation in the final results. This means that one can find some periodic orbits in a properly defined phase space. These periodic orbits are isolated, i.e. having the same conserved quantities in their neighbourhood, there is no other slightly deformed periodic orbits. However, by changing the parameters, hence, changing the conserved quantities only modestly, one periodic orbit grows to another like the periodic orbit is a cross section of cylinder in a 4-dimensional space. Since we have $\beta = 0$ in all our calculation, this space is a 3-dimensional one[9].

Those solutions of the equations in (21) are interesting for us which have the most resemblance to the experimental results, see Fig. 4 in the main text. From all the characteristics, we fix the period and the minimum and maximum values of ξ and η. We start with a 4N-dimensional initial condition $\mathscr{P}_z[\eta, \xi, T, \phi]$, where the arguments are 1-dimensional, N-element vectors of the values of the related nonlinear spectrum parameters at $z = 0$. Then we solve (21) for one period, T, and calculate the next $\mathscr{P}_n$. Periodicity of the results implies that the function[9]

$$\mathscr{F}(\mathscr{M}) = \mathscr{P}_0 - \mathscr{P}_z = 0,$$

where $\mathscr{M}$ is a subset of the 4N-dimensional space. To make the calculation simple and straightforward, $\mathscr{M}$ is chosen to be the period, and the minimum and maximum of the real and imaginary parts of the eigenvalue. Therefore, to find the parameters one only needs to solve $\mathscr{F}(\mathscr{M}) = 0$. To solve this equation, the SQP algorithm is used which is basically a variant of the Newton method. In essence, in a several iterations, the parameters get updated through $\mathscr{M}_{n+1} = \mathscr{M}_n + \Delta_n$ where the correction Δ_n is calculated from

$$\mathscr{F}(\mathscr{M}) + \mathscr{D}\Delta_n = 0$$

where $\mathscr{D}$ is the matrix of derivatives of $\mathscr{F}$ with respect to elements of $\mathscr{M}$. The solution of the above-mentioned problem yields the parameters of the assumed model in Eq. (17).

References

1. Sugavanam, S., Tarasov, N. & Churkin, D. V. Real-time intensity domain characterization of fibre lasers using spatio-temporal dynamics. *Appl. Sci. Basel* **6**, 65 (2016).

2. Hui, R. & O'Sullivan, M. *Fiber Optic Measurement Techniques* (Academic Press, 2009).

3. Churkin, D. V. et al. Stochasticity, periodicity and localized light structures in partially mode-locked fibre lasers. *Nat. Commun.* **6** (2015).

4. Ablowitz, M. J., Kaup, D. J., Newell, A. C. & Segur, H. The inverse scattering transform-fourier analysis for nonlinear problem. *Stud. Appl. Math.* **53** (1974).

5. Gardner, C. S., Greene, J. M., Kruskal, M. D. & Miura, R. M. Method for solving the kortweg-de vries equation. *Phys. Rev. Lett.* **19**, 1095–1097 (1967).

6. Zakharov, V. E. & Shabat, A. B. Exact theory of two-dimensional self-focusing and one-dimensional self-modulation of waves in nonlinear media. *JETP* **34**, 62–69 (1972).

7. Mertens, F. G., Quintero, N. R. & Bishop, A. R. Nonlinear schrödinger equation with spatiotemporal perturbations. *Phys. Rev. E* **81**, 016608 (2010).

8. Hasegawa, A. & Kodama, Y. *Solitons In Optical Communications* (Oxford University Press, 1995).

9. Flach, S. & Gorbach, A. V. Discrete breathers—advances in theory and applications. *Phys. Rep.* **467**, 1–116 (2008).
